# Implementation Challenges and Opportunities in Decentralized Methadone Service Delivery

**DOI:** 10.21203/rs.3.rs-7565402/v1

**Published:** 2025-10-11

**Authors:** Iddi Haruna Nkya, Feaven Gebrezgi, Adela Luswetula, Jessie Mbwambo, Haneefa T. Saleem

**Affiliations:** Muhimbili National Hospital; Johns Hopkins University; Johns Hopkins University; Muhimbili National Hospital; Johns Hopkins University

**Keywords:** medications for opioid use disorder, methadone maintenance therapy, decentralized service delivery, Africa

## Abstract

**Background:**

As the burden of opioid use disorder (OUD) increases in low- and middle-income countries, expanding access to medications for opioid use disorder, including methadone maintenance therapy, is essential. Decentralized models of methadone service delivery aim to improve treatment access, reduce costs for clients and burdens on health systems, and deliver more client-centered care. However, questions remain about the feasibility, acceptability, and implementation of decentralized models for methadone delivery, particularly in Africa. We qualitatively examined client and provider perspectives on decentralized methadone service delivery through satellite community health centers in Tanzania.

**Methods:**

We conducted a qualitative study with in-depth interviews among 10 providers and four focus groups with 40 participants (clients enrolled in OUD care, outreach workers, and treatment supporters) in Dar es Salaam, Tanzania. Through thematic analysis guided by the Consolidated Framework for Implementation Research (CFIR), we explored the experiences and perceptions of decentralized methadone services, including benefits, barriers, and recommendations.

**Results:**

Participants described key advantages of decentralization, such as reduced travel time and costs, improved access for clients with family or work obligations, and less congestion at central specialized opioid treatment clinic. However, they reported challenges across CFIR domains. Under intervention characteristics, clients perceived lower quality of care and less monitoring at satellite community health centers than at specialized opioid treatment clinics. Outer setting factors, such as privacy concerns and stigma, were perceived barriers to decentralized methadone delivery. Compared with specialized clinics, community health centers have fewer wrap-around services, reinforcing perceptions of poor quality of care. Individual characteristics, such as the risk of losing peer support networks and fears of drug testing, further discouraged clients from desiring to be transferred to community health centers. Finally, limited client involvement in planning and unclear communications were viewed as factors contributing to mistrust and resistance among clients to receive their methadone treatment at a community health center.

**Conclusions:**

Addressing community stigma, ensuring consistent service quality, supporting and enhancing clients’ social connections, and fostering transparent and participatory planning processes will be critical to effectively implement decentralized methadone service delivery.

## BACKGROUND

Methadone maintenance therapy is a critical component of opioid use disorder (OUD) management (1). In many African countries, methadone, which is classified as a controlled substance, is dispensed primarily through directly observed treatment in centralized, opioid treatment clinics, often located in hospitals in major urban centers. These centralized models have become a barrier to access, particularly for people unable to cover the costs of attending an authorized opioid treatment clinic daily and those who must travel long distances to receive care, particularly for those living outside of urban centers (2–5).

In response to these challenges, decentralized and differentiated models of methadone service delivery have been gaining traction globally. These models aim to improve access, reduce costs for clients, and deliver more client-centered care (6, 7). For example, the integration of methadone services into district-level health centers in Vietnam was shown to increase treatment uptake and reduce travel burdens (6). During the COVID-19 pandemic, Senegal piloted policies that allowed stable clients to take home methadone doses, ensuring continuity of care and enhancing treatment flexibility even with movement restrictions (8). Several high-income countries have adopted decentralized approaches to expand access to methadone and other medications for opioid use disorders. In the U.S., hub-and-spoke models have been used to extend the prescription of medications for opioid use disorder, specifically buprenorphine, in primary care settings (9–11). In France, Canada, Australia, and the United Kingdom, community pharmacies and general health clinics play a central role in delivering methadone and buprenorphine, reducing stigma and the burden on specialized facilities (12–16).

The evidence on decentralized methadone delivery in Africa remains limited. In South Africa, community-based harm reduction sites are exploring decentralized models, but widespread scale-up and formal regulation have yet to be achieved (17). Tanzania, the setting of the current study, started the first publicly funded opioid treatment program in mainland Africa, offering methadone maintenance therapy at the Muhimbili National Hospital (MNH) in Dar es Salaam starting in 2011 (18). Since then, methadone treatment has become available in several specialized clinics across the country, usually embedded within large, referral hospitals. Although buprenorphine delivery was piloted in 2021, access to buprenorphine remains limited in Tanzania. In 2022, the Tanzania Ministry of Health launched a differentiated service delivery model for opioid treatment similar to the hub-and-spoke model of care that has been successfully implemented in the U.S. (9–11) The hub-and-spoke network for opioid treatment in Tanzania includes bidirectional client transfers between specialized “hub” clinics at referral hospitals and community-based “spoke” primary care clinics, depending on client stability and preferences. In Tanzania, methadone can be prescribed by any medical doctor who is trained in medication-assisted therapy for OUD. As the demand for medications for opioid use disorder (MOUD), particularly methadone maintenance therapy, continues to grow across Africa, understanding the feasibility, acceptability, and implementation of decentralized models will be critical for informing accessible service delivery.

In this paper, we qualitatively examine client and provider perspectives on decentralized methadone service delivery through satellite community health centers in Dar es Salaam, Tanzania. By elucidating the experiences and perceptions of clients and providers, we hope to inform future strategies aimed at expanding and optimizing the accessibility of methadone services in Tanzania and other low- and middle-income countries exploring decentralized methadone service delivery.

## METHODS

For the present study, we use data collected from a study that examined barriers to and facilitators of methadone and HIV treatment adherence among clients attending an opioid treatment clinic (2, 19). Participants were recruited from a specialized opioid treatment clinic in Dar es Salaam. At recruitment, individuals were informed of the researchers’ institutions, the purpose and goals of the research study, and expectations for study participation. We conducted key informant interviews with 10 providers based at the central opioid treatment clinic who provided care for clients receiving methadone treatment, including medical doctors, nurses, pharmacists, social workers, and peer outreach workers. We also conducted four focus groups with 10 participants each. The focus group participants included 20 clients attending the opioid treatment clinic (two focus groups), 10 outreach workers (one focus group), and 10 treatment supporters (one focus group), who were mainly the partners and family members of clients enrolled in the opioid treatment program. We purposively sampled participants based on gender (for clients) and occupation (for providers and outreach workers). All participants provided informed consent before participation. Local research assistants, with bachelor’s level training in the social sciences and prior experience conducting qualitative research with people who use drugs, conducted the key informant interviews and focus groups face-to-face in Swahili via semi-structured guides at the study site, which was adjacent to the opioid treatment clinic or in the provider’s office. Research assistants did not have an existing relationship with participants before they participated in the study. The interviews and focus group guides included questions on the knowledge and attitudes of methadone delivered through satellite community health centers and the preferences of clients for receiving methadone at a specialized clinic versus a community health center (See guides in the Supplementary File). The interviews lasted approximately 1 hour, and the focus groups lasted 1.5–2 hours each. Participants were compensated for their time and contribution to the study.

The interviews and focus groups were digitally recorded, transcribed verbatim in Swahili, and translated into English. We developed a codebook that included a priori codes related to satellite perspectives and community health centers: criteria for transferring from a specialized clinic to the community health center, reasons for utilization, reasons for non-utilization, benefits, and challenges. Two members of the research team independently coded all transcripts via NVivo (Lumivero: Denver, Colorado). Discrepancies between the coders were resolved through discussion among the study team and consensus.

We organized emergent themes via the Consolidated Framework for Implementation Research (CFIR) (20). CFIR provides a comprehensive typology of multilevel factors that may influence the implementation of interventions across five domains: 1) intervention characteristics, 2) outer setting, 3) inner setting, 4) characteristics of individuals, and 5) process. Themes were mapped to CFIR constructs to facilitate interpretation of perceived barriers and facilitators to decentralized methadone service delivery. Data saturation was achieved on the themes presented. We did not conduct member checking with the participants.

Study protocols were approved by the Johns Hopkins Bloomberg School of Public Health and the Muhimbili University of Health and Allied Sciences institutional review boards, the Muhimbili National Hospital, and the National Health Research Ethics Committee at the National Institute for Medical Research in Tanzania.

## RESULTS

### Summary of findings

Our findings highlight both the advantages and challenges of decentralized methadone service delivery in Tanzania. The key themes that emerged from the data reveal how decentralization was perceived not only as improving methadone access and reducing client and provider burdens but also as introducing structural and social barriers that would need to be addressed to improve the acceptance of decentralization efforts among clients and providers.

### Intervention characteristics

#### Perceived lower quality of services at community health centers

Many participants perceived community health centers as lacking the same level of monitoring and accountability as the central, specialized opioid treatment clinic. This perceived discrepancy created doubts about the quality of care available at community health centers, with some clients viewing transfers to a community health center as a decrease in quality of care.

At [the central, specialized clinic], [the providers] are smart. They monitor you. If you take alcohol and attend there, they will just notice you, and actions will be taken… in those other [community health] clinics, they do not do such monitoring. (Client)

Clients and providers described rigorous monitoring at the central, specialized clinic as a key component of clients’ recovery journeys that reinforces adherence and deters potentially harmful substance use:

Respondent 1: There are some [clients] who like to come to Muhimbili [the specialized opioid treatment clinic] because they are assured that over here, they will complete their treatment, and it is because there are strict rules, and they are monitored accordingly. In addition, that is why they are happy coming to Muhimbili.

Respondent 2: That is what they say, that here they are very strict and so they are confident that they will complete their treatment. (Opioid treatment providers focus group)

#### Reduced clinic congestion and provider burden

Decentralization was also viewed as alleviating overcrowding at the specialized opioid treatment clinic, which treats up to 600 clients daily, on average. Providers and clients perceived decentralization, particularly the transfer of select clients to community health centers, as improving workflow and reducing wait times for clients:

It is true…to avoid congestion because at the beginning, most of us were drinking medicine here at [the specialized OTP clinic]… Now we are thankful for the small [health] center. (Client)

### Outer setting

#### Improved accessibility

The participants described how community health centers reduce the financial and logistical burden of accessing methadone treatment. For clients living far from the specialized clinic, decentralized methadone delivery was perceived as reducing travel time and costs, which participants reported as a significant barrier to treatment. The greater accessibility afforded by methadone dispensing in community health centers was viewed as particularly beneficial for clients with families and jobs, as it enables them to integrate methadone treatment more seamlessly into their daily lives:

[This decentralized methadone delivery approach] can help reduce travel costs, like bus fare costs. To reduce the cost of delay because someone might be staying at Tabata, then take his [methadone] medication at [the specialized clinic], so he will be delayed and so if there is a station there at Tabata, then it will be near, and he takes his medication there and continues with his activities. (Client)

The reduced distance and cost of travel were seen as critical to improving retention and adherence, particularly for clients from underserved areas:

The satellite clinics? They are good. It reduces the burden of fare because many [clients] lack the fare to come to the clinic. Some of them fail to receive treatment because of a lack of fare, so if the clinic is close to them, they will just easily go for treatment and continue their daily routines. (Opioid treatment provider)

#### Privacy and stigma

Privacy concerns emerged as a significant perceived barrier to engagement in decentralized services. Clients and peer outreach workers highlighted potential fears of being identified as someone with a history of heroin use as a deterrent to accepting to receive methadone at a community health center near one’s primary residence:

People want privacy. Yes…most of them want privacy… There are people who are intelligent, who own shops, and who have their own offices. You see? So, there is someone who stays at Mbagala and has his own shop at Kariakoo…no one knows. He comes, takes his medication, and off, he leaves. But you are asking him to stay at Mbagala; it is as if you are exposing him. (Peer outreach worker)

An opioid treatment provider echoed this perception: “I spoke to most of them [clients], and they said they don’t want the surrounding community to know that they are addicted to drugs. He does not want the community, that his friends know. When they know that they are on methadone, they automatically know that they abuse drugs. So, that’s the biggest reason.”

#### Inner setting

The participants also described how differences in staffing levels and available services between the specialized opioid treatment clinic and the satellite community health centers shaped perceptions of decentralized care. While the specialized clinic offered more comprehensive wrap-around services, including counseling, social support, and frequent monitoring, community health centers dispensing methadone were viewed as having fewer resources and less specialized staff capacity to address the complex needs of clients:

Some are on high [methadone] doses and have many problems. For example, those who have TB [tuberculosis] and are on anti-TB [medications], so they need to see a doctor. But some [clients] have other diseases and need to see a doctor. In those satellites [community health centers], there are no specialist doctors, and some of the equipment is not there, so some services are not offered there…they don’t have enough professionals. (Opioid treatment provider)

These aspects of the inner setting at community health centers dispensing methadone contributed to perceptions of lower service quality, as described under the intervention characteristics above, and revealed how the community health center environment, especially compared with more specialized services offered at the specialized clinic, and the design of the decentralized model were closely intertwined in shaping participants’ views. These perceived deficiencies at community health centers were reported as contributing to the hesitation of clients to transfer to community health centers, particularly for those clients who valued the integrated support available at the central, specialized opioid treatment clinic.

### Individual characteristics

#### Loss of social support networks

The participants expressed the reluctance of clients to transfer because doing so would disrupt valued peer relationships that they feel support recovery. Clients were described as forming close-knit relationships with peers at the specialized clinic. Participants described how transferring to a community health center can disrupt these relationships:

[S]ome fear that once they go [to a community health center to continue their methadone treatment], they would break up with their friends here. That’s also another reason contributing to holding them up…You may find a person having six friends here although they are not staying together but just meet up here and after the treatments, they sit there outside drinking porridge. So, when today you tell the person to move to satellite clinics, it means he or she will not meet up with his or her friends; thus, they also fear this. (Peer outreach worker)

#### Fear of drug testing

Mandatory urine testing to determine eligibility for transfer to a community health center for methadone dispensing deterred some clients from considering decentralized services. Fear of testing positive heroin or other drugs was viewed as creating resistance among clients to the transfer process. As one peer outreach worker said, “They are afraid to get tested because they see they will be discovered with their habit of drug use… so they prefer to stay [at the specialized clinic].”

### Process

#### Lack of client involvement in planning and decision-making

Participants, particularly peer outreach workers and clients, consistently described a top-down approach to identifying community health centers to dispense methadone, which did not engage clients or peer outreach worker. This exclusion of clients and peer outreach workers from the planning process was perceived as resulting in dissatisfaction with the community health centers selected to dispense methadone.

The issue is that the time when they were doing the feasibility study of the satellite clinics [at community health centers], it was like it was decided from the top level without involving the bottom level. It was supposed to be the clients deciding where the locations should be to simplify reaching [the satellite, community health center]. (Peer outreach worker)

#### Limited communication and misconceptions

Limited communication about the purpose and criteria for transfers was perceived as contributing to mistrust and misunderstandings among clients. Participants emphasized the need for better communication to address misconceptions about transfers to community health centers for methadone dispensing. Clients and peer outreach workers described a belief among clients that transfers are punitive and highlighted clients’ limited understanding of the eligibility criteria and purpose of these transfers:

I think there was insufficient information. That’s why a client is told that their center will be at Mbagala [a community health center], but they refuse to go because they don’t have sufficient information…You will hear them say, “If you now misbehave at Muhimbili, your punishment is that you will be moved to Segerea [a community health center near a prison]. So, they see the satellite clinic [community health center] as punishment. (Peer outreach worker)

Some of the peer outreach workers suggested involving outreach workers in demand generation efforts to promote community health centers for methadone dispensing:

We should come up with a motto if we want them to attend the satellites… If we give out enough information, I know my people, they can do it, and they can change and decide to go there. (Peer outreach worker)

## DISCUSSION

This study examined client and provider perspectives on decentralized methadone delivery through satellite community health centers in Tanzania. We identified how factors across the intervention characteristics, i.e., the decentralized model, the outer setting, the inner setting, individual characteristics, and the process, influenced perceptions and experiences of decentralized methadone delivery. This approach provided a structured understanding of the multilevel barriers and facilitators shaping implementation, including service quality concerns, stigma, client readiness, and limited participatory planning.

Decentralized methadone delivery through community health centers was viewed as alleviating congestion and reducing client and provider burden, which was viewed positively by clients, peer outreach workers, and providers. These reported advantages of decentralization mirror global findings from Vietnam, the U.S., and elsewhere, where decentralized MOUD models have reduced client burden (6, 21, 22). However, perceived differences in service quality and client monitoring shaped perceptions of clients’ willingness to transfer to community health centers to receive methadone. While the decentralized model was widely viewed as improving access, many participants described community health centers that dispensed methadone as offering less rigorous oversight and suboptimal quality of care than the specialized opioid treatment clinics embedded in referral hospitals.

The outer setting, including community stigma toward people with a history of drug use and fears of compromised privacy, emerged as a critical perceived barrier to decentralized methadone delivery. The participants highlighted that receiving methadone closer to home could increase the risk of social judgment and discrimination, echoing research findings from other settings that the decentralization of methadone treatment to community settings can inadvertently expose clients to stigma and social harm if confidentiality and respectful communication are not adequately maintained (23).

Process factors, including the lack of client involvement in service planning and poor communication, were identified as barriers to implementing decentralized methadone delivery. Clients and peer outreach workers reported feeling excluded from decisions around clinic locations and transfers and were often misinformed about the purpose of decentralization, with many misinterpreting transfers to community health centers for methadone treatment as punitive. These findings suggest that transparent messaging and client-centered approaches to designing key aspects of decentralized service delivery are essential to ensure client acceptance and buy-in. Studies from Ireland and South Africa have demonstrated that including clients in informing opioid treatment policies and practices can improve responsiveness, reduce barriers, and foster trust in treatment delivery (24, 25).

At the individual client level, clients’ readiness for decentralized care, peer support networks, and fears of mandatory drug testing also shaped perceptions. Many clients and peer outreach workers described how transferring to community health centers for methadone treatment would disrupt important peer support networks established at the specialized opioid treatment clinic. This highlights the need to balance convenience with preserving supportive social environments, which could support treatment retention and recovery. The value of social cohesion among clients in facilitating methadone treatment retention has been previously reported in South Africa (26) and should be considered in decision-making in decentralization efforts. Fear of mandatory urine drug testing for transfers and unclear eligibility criteria was viewed as further hindering willingness among clients to transfer their methadone treatment to community health centers. Similar challenges have been documented in the U.S., Canada, and Vietnam, where rigid protocols and a lack of communication disrupted the continuity of care for clients (7, 12, 27). Engaging clients early on in planning and decision-making processes can help to predict and mitigate potential implementation challenges.

Findings from this study reinforce the notion that successful decentralization requires more than the physical relocation of services; it demands intentional strategies to achieve client buy-in, address stigma, ensure service quality, and support clients’ social needs. Global evidence supports the integration of stigma-reduction strategies and peer support to improve retention and foster community acceptance in opioid treatment programs (6, 23, 28). As countries explore strategies to expand access to methadone treatment, the introduction and scale-up of buprenorphine, particularly because of its safety profile and suitability for community-based delivery, represents an important future direction. However, access to buprenorphine remains limited in many African settings due to regulatory, supply chain, and cost issues, underscoring the need for coordinated policy and system-level investments to diversify and strengthen treatment options.

This study has several limitations. First, findings are based on one opioid treatment network and may not reflect broader experiences. Second, we did not interview healthcare providers at satellite community health centers delivering methadone. Because of this, we did not assess community health center provider readiness or training, which are known to impact the quality of care (7). Third, the main focus of the guide was not to explore perspectives on decentralized methadone service delivery, so we did not set out to explore all CFIR domains. However, our thematic analytic revealed themes that aligned to the five CFIR domains. Future research, particularly as decentralized methadone service delivery efforts are scaled, would benefit from study designs grounded implementation science frameworks. Nonetheless, these findings contribute to a growing evidence base on implementation strategies to decentralize methadone delivery in low- and middle-income countries.

## CONCLUSIONS

This study provides valuable insights into the experiences and perceptions of clients and providers regarding decentralized methadone delivery in Tanzania. The findings underscore the importance of addressing community stigma, ensuring consistent service quality, supporting and enhancing clients’ social connections, and fostering transparent, participatory implementation planning processes.

## Supplementary Material

Supplementary Files

This is a list of supplementary files associated with this preprint. Click to download.

• FGDguideBMCHealthServices24.09.2025.docx

• IDIguideBMCHealthServices24.09.2025.docx

## Data Availability

The datasets used and/or analyzed during the current study are available from the corresponding author on reasonable request.

## Abbreviations

CFIR: Consolidated Framework for Implementation Research
HIV: Human immunodeficiency virus
MOUD: Medications for opioid use disorder
OTP: Opioid treatment program
OUD: Opioid use disorder
